# Supplementary material for: Deep learning methods for drug response prediction in cancer: Predominant and emerging trends
Source: Front Med (Lausanne). 2023 Feb 15;10:1086097. doi: 10.3389/fmed.2023.1086097 (PMC9975164; doi:10.3389/fmed.2023.1086097)
Supplement: Supplementary file 1 [file Data_Sheet_1.PDF]

Supplementary material

Table S1. A list of drug response prediction models that utilize neural networks. The data were collected until August of 2022, considering only peer-reviewed publications since 2013.

Paper	Model	Framework	Methods	Cancer features	Drug features	Response cell line	Response PDX	Response patient	Evaluation scheme
Hostallero et al. 2022	BiG-DRP	PyTorch	GNN	GE	DD	IC50	NA	IC50-to-Bin	Cancer-blind, Mixed-set
Yan et al. 2022	DGSDRP	PyTorch	1D-CNN, GNN, bRNN	Mu	MG	IC50	NA	NA	Cancer-blind, Drug-blind, Mixed-set
Xia et al. 2022	UnoMT	TF1	RC, AE, MTL	GE	DD, FP	AUC	NA	NA	Cross-dataset, Mixed-set
Wang et al. 2022	MvMo	PyTorch	MVL	GE, Methyl, Mu	MG	IC50	NA	NA	Cancer-blind, Drug-blind, Mixed-set
Su et al. 2022	SRDFM	TF1 (w/o Keras)	DeepFM	GE	FP	Rank	NA	NA	Cross-dataset, Drug-blind
Pu et al. 2022	CancerOmics Net	PyTorch	Att, GNN	GE	KIP	GR	NA	NA	Cancer-blind
Prasse et al. 2022	Conv NN	TF2	1D-CNN, TL	GE	SMILES	IC50	CTR	NA	Cancer-blind, Cross-dataset, Drug-blind
Prasse et al. 2022	None	TF2 (w/o Keras)	1D-CNN, Att, RC	GE	SMILES	Rank	NA	NA	Cancer-blind
Peng et al. 2022	MOFGCN	PyTorch	GNN	CNV, GE, Mu	FP	Bin-enc-IC50	NA	NA	Cancer-blind, Drug-blind, Mixed-set
Nguyen et al. 2022	GraOmicDRP	PyTorch	1D-CNN, GNN	CNV, GE, Methyl, Mu	MG	IC50, Bin-enc-IC50	NA	NA	Cancer-blind, Drug-blind, Mixed-set
Ma et al. 2022	DualGCN	TF1	GNN	CNV, GE	MG	IC50	NA	IC50-to-Bin	Cross-dataset, Mixed-set
Liu et al. 2022	PPORank	NA	RNN, RL	GE, Methyl	NA	Rank	NA	Rank	Cancer-blind, Cross-dataset
Jiang et al. 2022	DeepTTA	PyTorch	Tran	GE	FP	IC50-to-Bin	NA	NA	Cancer-blind, Drug-blind, Mixed-set
Chu et al. 2022	GraTransDRP	PyTorch	1D-CNN, GNN, Tran	CNV, GE, Mu	MG	IC50	NA	NA	Cancer-blind, Drug-blind, Mixed-set
Zuo et al. 2021	SWnet	PyTorch	1D-CNN, GNN, Att, MTL	GE, MU	FP, MG	IC50	NA	NA	Cancer-blind, Mixed-set
Zhu et al. 2021	TGSA	PyTorch	GNN, MTSPT, SSPT	CNV, GE, Mu	FP, MG	IC50	NA	NA	Cancer-blind, Disjoint-set, Drug-blind, Mixed-set
Zhu et al. 2021	IGTD	TF1	2D-CNN	GE	DD	AUC	NA	NA	Mixed-set

Zhang et al. 2021	ConsDeepSignaling	TF1	Int. NN	CNV, GE	NA	AUC	NA	NA	Cancer-blind
Tang et al. 2021	PathDSP	PyTorch	FC-NN (EI)	CNV, GE, Mu	FP	IC50	NA	NA	Cancer-blind, Cross-dataset, Drug-blind
Snow et al. 2021	BDKANN+	TF2	Int. NN	GE	NA	AAC	NA	NA	Cancer-blind, Cross-dataset
Sharifi-Noghabi et al. 2021	Velodrome	PyTorch	TL, AL	GE	NA	AAC	Bin	Bin	Cancer-blind, Cross-dataset
Peres da Silva et al. 2021	TUGDA	PyTorch	AE, AL, BNN, TL, MTL	GE	NA	IC50	CTR	Bin	Cancer-blind, Cross-dataset
Partin et al. 2021	None	TF2	FC-NN (LI)	GE	DD	AUC	NA	NA	Mixed-set
Park et al. 2021	Super.FELT	PyTorch	FC-NN (LI)	CNV, GE, Mu	NA	Bin-enc-IC50	Bin	Bin	Cancer-blind, Cross-dataset
Nguyen et al. 2021	GraphDRP	PyTorch	1D-CNN, GNN	CNV, Mu	MG	IC50	NA	NA	Cancer-blind, Drug-blind, Mixed-set
Malik et al. 2021	None	Matlab	FC-NN (EI)	CNV, GE, Methyl, Mu	NA	IC50-to-Bin	NA	NA	Cancer-blind
Ma et al. 2021	TCRP	PyTorch	TL, ML	GE, Mu	NA	AUC	CTR	NA	Cancer-blind, Cross-dataset
Liu et al. 2021	GraphCDR	PyTorch	1D-CNN, Att, GNN, CL	GE, Methyl, Mu	MG	Bin-enc-AAC, Bin-enc-IC50	NA	NA	Cross-dataset, Mixed-set
Koras et al. 2021	DEERS	PyTorch	AE	GE, Mu	KIP	AUC, IC50	NA	NA	Cancer-blind, Cross-dataset, Drug-blind, Mixed-set
Kim et al. 2021	DrugGCN	TF1 (w/o Keras)	GNN	GE	NA	AUC, IC50	NA	NA	Cancer-blind
Jin et al. 2021	HiDRA	TF1	Att	GE	FP	IC50	NA	NA	Cancer-blind, Cross-dataset, Drug-blind, Mixed-set
Jiang et al. 2021	DrugOrchestra	PyTorch	MTL	GE	Pre-trained MG	IC50	CTR	NA	Cross-dataset, Drug-blind, Mixed-set
Jia et al. 2021	VAEN	TF1	VAE	GE	NA	AAC, IC50	NA	AA-to-Bin	Cancer-blind, Cross-dataset
Feng et al. 2021	AGMI	PyTorch	Att, GNN	CNV, GE, Mu	MG	IC50	NA	NA	Mixed-set
Emdadi et al. 2021	Auto-HMM-LMF	NumPy	AE	CNV, GE, Mu, TT	FP	Bin-enc-IC50	NA	NA	Drug-blind, Mixed-set
Dong et al. 2021	None	PyTorch	JT-VAE, VAE	GE	MG	IC50	NA	NA	Mixed-set
Zhu et al. 2020	tDNN	TF1	TL, EL	GE	DD	AUC	NA	NA	Cancer-blind, Cross-dataset, Drug-blind, Mixed-set
Tao et al. 2020	CADRE	PyTorch	Att, CF	GE	NA	Bin-enc-AAC	NA	NA	Cancer-blind

Sharifi-Noghabi et al. 2020	AITL	PyTorch	TL, MTL	GE	NA	IC50	Bin	Bin	Cancer-blind, Cross-dataset
Liu et al. 2020	DeepCDR	TF1	1D-CNN, GNN	GE, Methyl, Mu	MG	IC50	NA	Bin	Cancer-blind, Cross-dataset, Disjoint-set, Drug-blind, Mixed-set
Li et al. 2020	MFNN	TF1	1D-CNN, RNN	GE, DD	DD	IC50	NA	NA	Mixed-set
Kuenzi et al. 2020	DrugCell	PyTorch	Int. NN	Mu, FP	FP	AUC	AUC-to-Bin	AUC-to-Bin	Cross-dataset, Mixed-set
Deng et al. 2020	pathDNN	PyTorch	Int. NN	GE	NA	AAC	NA	NA	Cancer-blind, Cross-dataset, Mixed-set
Daoud et al. 2020	Q-Rank	R	RL	GE, Methyl, Mu, RPPA	DD	GI50-to-Bin	NA	NA	Cancer-blind, Mixed-set
Choi et al. 2020	RefDNN	TF1 (w/o Keras)	FC-NN (EI)	GE	FP	Bin-enc-IC50	NA	NA	Cancer-blind, Drug-blind, Mixed-set
Bazgir et al. 2020	REFINED	TF2	2D-CNN	GE	DD	IC50, GI50, Bin-enc-GI50	NA	NA	Mixed-set
Ahmed et al. 2020	None	PyTorch	GNN	GE	NA	AUC	NA	NA	Cancer-blind
Zhao et al. 2019	None	TF1	FC-NN (EI)	GE	NA	AAC-to-mc	NA	NA	Cancer-blind
Xu et al. 2019	AutoBorutaRF	H2O.ai	AE	CNV, GE, Mu	NA	Bin-enc-IC50	NA	NA	Cancer-blind
Sharifi-Noghabi et al. 2019	MOLI	PyTorch	FC-NN (LI)	CNV, GE, Mu	NA	Bin-enc-IC50	Bin	Bin	Cancer-blind, Cross-dataset
Sakellaropoulos et al. 2019	---	H2O.ai	FC-NN (EI)	GE	NA	IC50-to-Bin	NA	Bin	Cancer-blind, Cross-dataset
Rampášek et al. 2019	Dr.VAE	PyTorch	VAE	GE	NA	Bin-enc-AAC	NA	NA	Cancer-blind
Manica et al. 2019	MCA	TF1 (w/o Keras)	1D-CNN, Att, bRNN, RC	GE	SMILES	IC50	NA	NA	Disjoint-set, Mixed-set
Liu et al. 2019	tCNNS	TF1 (w/o Keras)	1D-CNN	CNV, Mu	SMILES	IC50	NA	NA	Cancer-blind, Drug-blind, Mixed-set
Li et al. 2019	DeepDSC	TF1	AE	GE	FP	IC50	NA	NA	Cancer-blind, Drug-blind, Mixed-set
Joo et al. 2019	DeepIC50	TF1	1D-CNN	Mu	DD, FP	mc-enc-IC50	NA	mc	Cross-dataset, Mixed-set
Chiu et al. 2019	DeepDR	TF1	AE	GE, Mu	NA	IC50	NA	NA	Cancer-blind
Oskooei et al. 2018	PaccMann	TF1 (w/o Keras)	1D-CNN, Att, bRNN	GE	SMILES	IC50	NA	NA	Disjoint-set, Mixed-set
Ding et al. 2018	---	Matlab	AE	CNV, GE, Mu	NA	Bin-enc-AAC	NA	NA	Cancer-blind

Chang et al. 2018	CDRscan	TF1	1D-CNN, EL	Mu	FP	IC50	NA	NA	Mixed-set
Menden et al. 2013	---	Java	FC-NN (EI)	CNV, Mu	DD	IC50	NA	NA	Cross-dataset, Mixed-set

Abbreviations

NN modules

1. NN: neural network
2. 1D-CNN and 2D-CNN: one- and two-dimensional convolutional NN
3. Att: attention modules
4. GNN: graph NN
5. Inter: interpretable NN
6. RNN: recurrent NN
7. RC: residual connections

Learning schemes

1. AE: autoencoder
2. AL: adversarial learning
3. BNN: Bayesian NN
4. CF: collaborative filtering
5. CL: contrastive learning
6. DeepFM: deep factorization machine
7. EL: ensemble learning
8. ML: meta learning
9. MVL: multi-view learning
10. MTL: multi-task learning
11. MTSPT: multi-task supervised pre-training
12. RL: reinforcement learning
13. SSPT: self-supervised pre-training
14. TL: transfer learning
15. Tran: transformer

Cancer features

1. CNV: copy number variation
2. GE: gene expression
3. Methyl: methylation
4. Mu: mutation
5. RPPA: reverse phase protein arrays
6. TT: tissue type

Drug features

1. SMILES: simplified molecular-input line-entry system
2. DD: drug descriptors
3. FP: drug fingerprints
4. MG: molecular graphs
5. KIP: drug kinase inhibition profiles

Response

1. AAC: area above the dose response curve
2. AUC: area under the dose response curve
3. Rank: ranking
4. IC50-to-Bin: IC50 converted to binary
5. AUC-to-MC: AUC converted to multi-class
6. GI50-to-bin: GI50 converted to binary
7. Bin-enc-IC50: binary encoded IC50
8. Bin-enc-AUC: binary encoded AUC
9. Bin-enc-AAC: binary encoded AAC
10. Bin-enc-GI50: binary encoded GI50
11. MC-enc-IC50: multi-class encoded IC50
12. CTR: continuous tumor response
